# Faux anévrisme artériel traumatique intracrânien

**DOI:** 10.11604/pamj.2015.20.158.5461

**Published:** 2015-02-19

**Authors:** Jawad Laaguili, Abad Cherif El Asri, Miloud Gazzaz, Moulay Rachid El Hassani, Brahim El Mostarchid

**Affiliations:** 1 Service de Neurochirurgie de l'Hôpital Militaire Mohammed V de Rabat, Maroc; 2 Service de Radiologie de l'Hôpital des Spécialités, Rabat, Maroc

**Keywords:** Traumatisme crânien, anévrysmes traumatiques, angiographie, traitement endovasculaire, head injury, traumatic aneurysms, angiography, endovascular treatment

## Abstract

Nous rapportons un cas d'anévrysme post-traumatique de l'artère carotide interne chez un enfant de 11 ans, ayant présenté une ophtalmoplégie droite. Un faux anévrisme de la carotide interne droite a été diagnostiqué par angiographie cérébrale. Le malade a bénéficié d'un traitement endovasculaire et l’évolution fut favorable. Malgré leur rareté, le diagnostic des anévrysmes post traumatiques devrait être évoqué chez tout traumatisé crânien en cas d'aggravation clinique secondaire, afin de réaliser une exploration angiographique avant d'envisager un traitement radical soit chirurgical soit par voie endovasculaire.

## Introduction

Les anévrismes post-traumatiques représentent moins de 1% des anévrismes artériels intracrâniens [1, 2], ils sont plus fréquents chez l'enfant [2, 3]. Leur évolution imprévisible, comporte un risque de rupture fatale. Le rôle de l'imagerie est primordial aussi bien pour le diagnostic que pour un éventuel geste thérapeutique endovasculaire. Nous rapportons un cas d'anévrisme post-traumatique de l'artère carotide interne.

## Patient et observation

**Figure 1:**
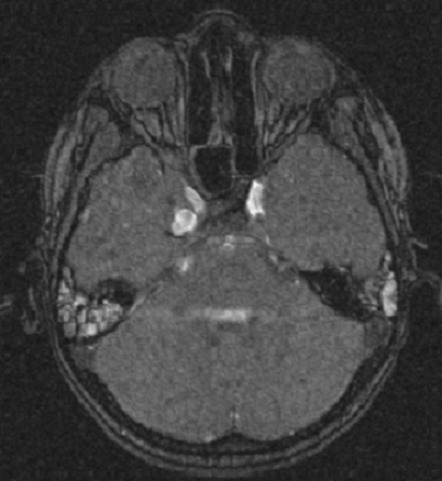
IRM cérébrale coupe axiale en T1 objective une lésion intra caverneuse droite en hyper signal, faisant suspecter une origine vasculaire

**Figure 2:**
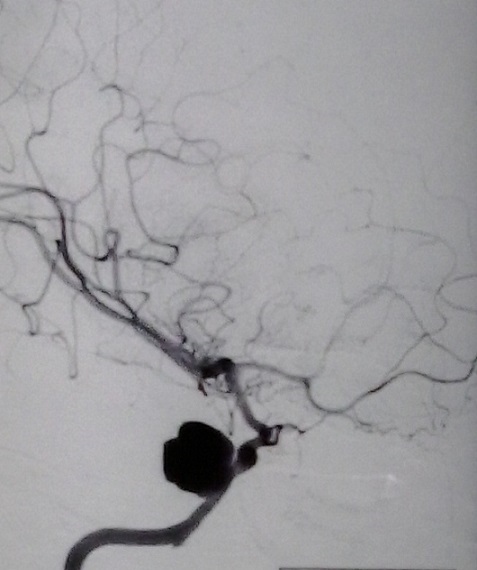
Angiographie de l'artère carotide interne droite, incidence de profil. Image d'addition à contours irréguliers au niveau du siphon carotidien

**Figure 3:**
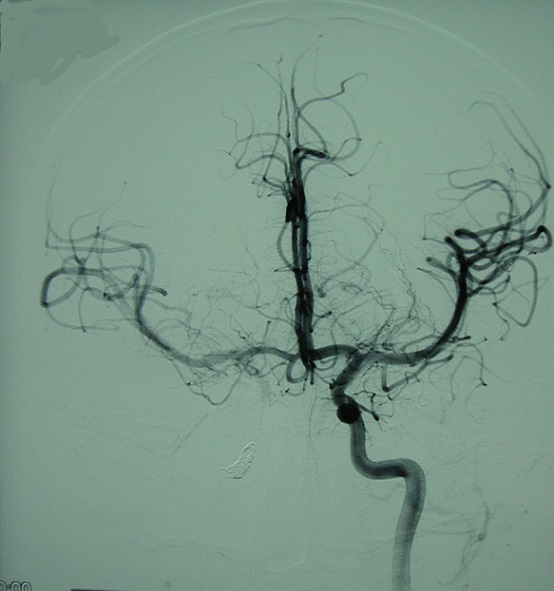
Angiographie de l'artère carotide interne gauche après occlusion de l'artère carotide droite. Incidence de face. Bonne prise en charge du territoire carotidien droit par la communicante antérieure et postérieure

Enfant de 11 ans, qui a été victime d'un traumatisme crânien grave suite a un accident de la voie publique, À l'admission, le patient était dans le coma, ayant nécessité un séjour en réanimation, après reprise de l’état de conscience, le patient a présenté une diplopie horizontale. L'examen neurologique a objectivé une ophtalmoplégie droite avec atteinte du III, IV et VI nerfs crâniens associé à une hypoesthésie dans le territoire du V1 droit, par ailleurs, on note l'absence d'une exophtalmie pulsatile. Le scanner cérébral a révélé une fracture de l’étage antérieur irradiant vers le rocher droit avec comblement des cellules mastoïdiennes. L'IRM cérébrale objective une lésion intra caverneuse droite hypersignal T1, faisant suspecter une origine vasculaire (Figure 1). Devant ce tableau de syndrome du sinus caverneux et des données de l'imagerie, une angiographie cérébrale demandée objectivant l'existence d'un faux anévrisme du segment intracaverneux de la carotide interne droite (Figure 2). Un traitement endovasculaire a été réalisé avec mise en place de 5 coils ayant permis l'exclusion de la carotide interne droite. Le contrôle final montre la prise en charge du territoire carotidien droit via les artères communicantes antérieure et postérieure (Figure 3). Les suites étaient simples avec bonne amélioration clinique et régression du syndrome du sinus caverneux.

## Discussion

Sur le plan anatomopathologique, les anévrismes post-traumatiques sont des « faux anévrismes » résultant d'une rupture de toutes les couches de la paroi artérielle. L'hémorragie ne peut se propager, l'hémostase se faisant au contact des structures adjacentes sous forme d'un conglomérat de fibrine et de macrophages réalisant une fausse paroi. Sous l'effet de la pression artérielle se crée une fausse lumière anévrismale [1, 2]. Ces lésions résultent d'un traumatisme mural direct, ou d'un mouvement de torsion translation entraînant des phénomènes de cisaillements des parois artérielles [1, 4]. Les lésions de la base du crâne sont responsables d'anévrismes de l'artère carotide interne et plus rarement d'anévrismes vertébrobasilaires [5]. Les vaisseaux de la base sont endommagés par les éléments intracrâniens susceptibles d’être vulnérants: arête sphénoïdale, bord libre de la tente du cervelet, bord libre de la faux du cerveau. L'hémorragie intracrânienne représente le mode de manifestation clinique le plus fréquent avec un taux entre 41 et 57% selon les séries [6].

L'examen scannographique reste un examen performant de première intention pour établir le diagnostic. L'angiographie reste indispensable, mais l'absence d'anomalies vasculaires sur un bilan réalisé précocement après le traumatisme n'exclut pas un développement anévrismal ultérieur [7]. L’évolution de ces anévrismes est caractérisée par une augmentation progressive de leur taille objectivée par les contrôles angiographiques en absence de traitement. Mais une thrombose spontanée de l'anévrysme a été décrite dans quelques cas. Le traitement est fonction de la taille de l'anévrysme, de sa localisation, de la présence éventuelle d'un collet mais aussi de l’état clinique du malade [3, 6]. Ce traitement peut être réalisé soit par abord chirurgical, soit par voie endovasculaire. Les anévrysmes cérébraux traumatiques non traités ont un pronostic sombre; la mortalité se situe entre 18% et 54% [6]. Cette mortalité est plus élevée chez les patients pour qui le diagnostic est porté après une hémorragie sous-arachnoidienne [1].

## Conclusion

Malgré leur rareté, les anévrismes intracrâniens post traumatiques devraient être suspectés chez un traumatisé crânien, récent ou ancien, même mineur présentant une aggravation clinique secondaire, afin de réaliser une exploration angiographique en urgence, avant d'envisager un traitement radical soit chirurgical soit par voie endovasculaire.
